# Crystal structure of a layered phosphate molybdate K_2_Gd(PO_4_)(MoO_4_)

**DOI:** 10.1107/S2056989023011106

**Published:** 2024-01-05

**Authors:** Valeriia Zozulia, Kateryna Terebilenko, Artem Voinalovych, Vadim Potaskalov, Mykola Slobodyanik

**Affiliations:** a Taras Shevchenko National University of Kyiv, Volodymyrska St. 64, Kyiv 01601, Ukraine; b Department of General and Inorganic Chemistry, National Technical University of Ukraine "Igor Sikorsky Kyiv Polytechnic Institute", 37 Prospect Beresteiskyi, 03056 Kyiv, Ukraine; Harvard University, USA

**Keywords:** crystal structure, molybdate, phosphate, gadolinium, triangular dodeca­hedron

## Abstract

The title compound dipotassium gadolinium(III) phosphate(V) molybdate(VI), K_2_Gd(PO_4_)(MoO_4_), was synthesized from a high-temperature melt starting from GdF_3_ as a source of gadolinium. Its structure is isotypic with other *M* ^I^_2_ *M* ^III^(*M* ^VI^O_4_)(PO_4_) compounds, where *M* ^I^ = Na, K or Cs, and *M* ^III^ = rare-earth cation, *M* ^VI^ = Mo or W. The three-dimensional framework is built up from [Gd(PO_4_)(MoO_4_)] anionic sheets, which are organized by adhesion of [GdPO_4_] layers and [MoO_4_] tetra­hedra stacked above and below these layers. The inter­stitial space is occupied by K cations having eightfold oxygen coordination. The polyhedron of GdO_8_ was estimated to be a triangular dodeca­hedron by the continuous shape measurement method.

## Abstract 2

Dipotassium gadolinium(III) phosphate(V) molybdate(VI), synthesized from a high-temperature melt starting from GdF_3_ as a source of gadolinium, has a structure that is isotypic with other *M* ^I^_2_ *M* ^III^(*M* ^VI^O_4_)(PO_4_) compounds, where *M* ^I^ = Na, K or Cs, and *M* ^III^ = rare-earth cation, *M* ^VI^ = Mo or W. The three-dimensional framework is built up from [Gd(PO_4_)(MoO_4_)] anionic sheets, which are organized by adhesion of [GdPO_4_] layers and [MoO_4_] tetra­hedra stacked above and below of these layers, and the inter­stitial space is occupied by K cations having eightfold oxygen coordination.

## 1. Chemical context

Layered phosphate(V) molybdates(VI) *M* ^I^_2_ *M* ^III^(*M* ^VI^O_4_)(PO_4_) comprising an alkali metal and a rare-earth metal *M* ^III^ such as Sm (Zhao *et al.*, 2009 ▸), Eu (Terebilenko *et al.*, 2022 ▸), Y (Zhang *et al.*, 2016 ▸) or Bi (Grigorjevaite *et al.*, 2020 ▸) are considered to be promising luminescent materials (Guo *et al.*, 2019 ▸). The initial structural models of this group of compounds, Na_2_Y(PO_4_)(MoO_4_), were monoclinic, space group *C*2/*c*, as described by Ben Amara & Dabbabi (1987 ▸). Subsequent work determined that the material crystallizes in an ortho­rhom­bic system, space group *Ibca* (Marsh, 1987 ▸). The discovery of K_2_Bi(PO_4_)(MoO_4_) by Zatovsky *et al.* (2006 ▸) opened a new group of luminescent materials that are isostructural to Na_2_Y(PO_4_)(MoO_4_) and have high color purity and quantum yield (Grigorjevaite & Katelnikovas, 2016 ▸).

In the case of Rb_2_Bi(PO_4_)(MoO_4_):Eu^3+^ powders, the quantum efficiency has been shown to reach *ca* 100% for the Rb_2_Bi_0.5_Eu_0.5_(PO_4_)(MoO_4_) phosphor (Grigorjevaite & Katelnikovas, 2016 ▸). High color purity and emission spectra peculiarities make these compounds attractive for red-component design in near-UV LED-driven solid-state light sources (Zozulia *et al.*, 2023 ▸). One of the main disadvantages of these luminescence hosts is the relatively high activator content needed (from 50 to 75%) to reach a high quantum efficiency (Grigorjevaite & Katelnikovas, 2016 ▸). Different strategies have been applied to improve the luminescence performance and lower the luminescent dopant content, including rare-earth co-doping (Naidu *et al.*, 2012 ▸) and anion modifications (Guo *et al.*, 2019 ▸). To tune the luminescence properties of these phosphors, the quest for new representatives of this group of compounds can shed light on the development of new phosphors based on them.

## 2. Structural commentary

**Figure 1:**
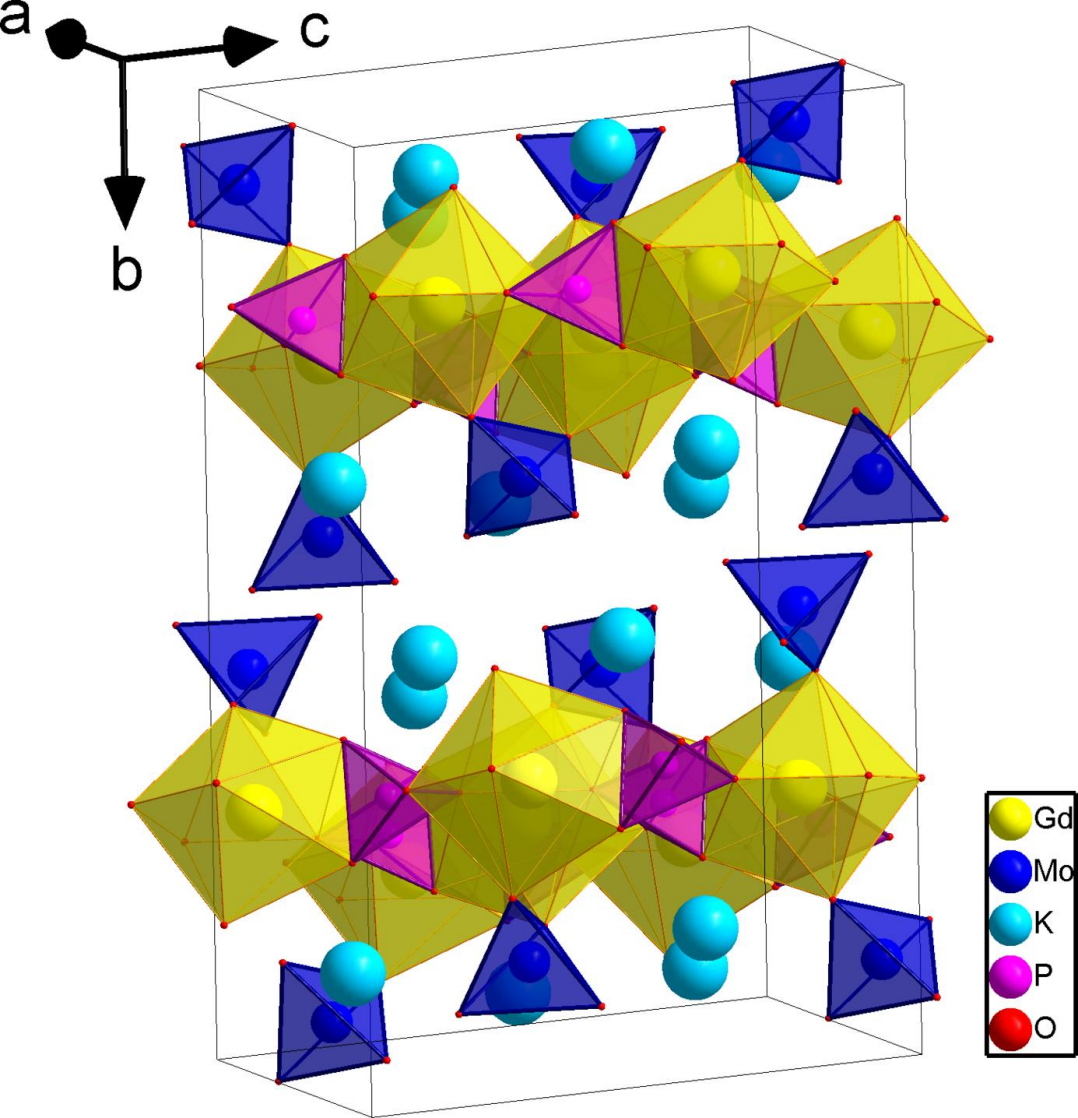
Representation of the unit-cell content of K_2_Gd(PO_4_)(MoO_4_).

**Figure 2:**
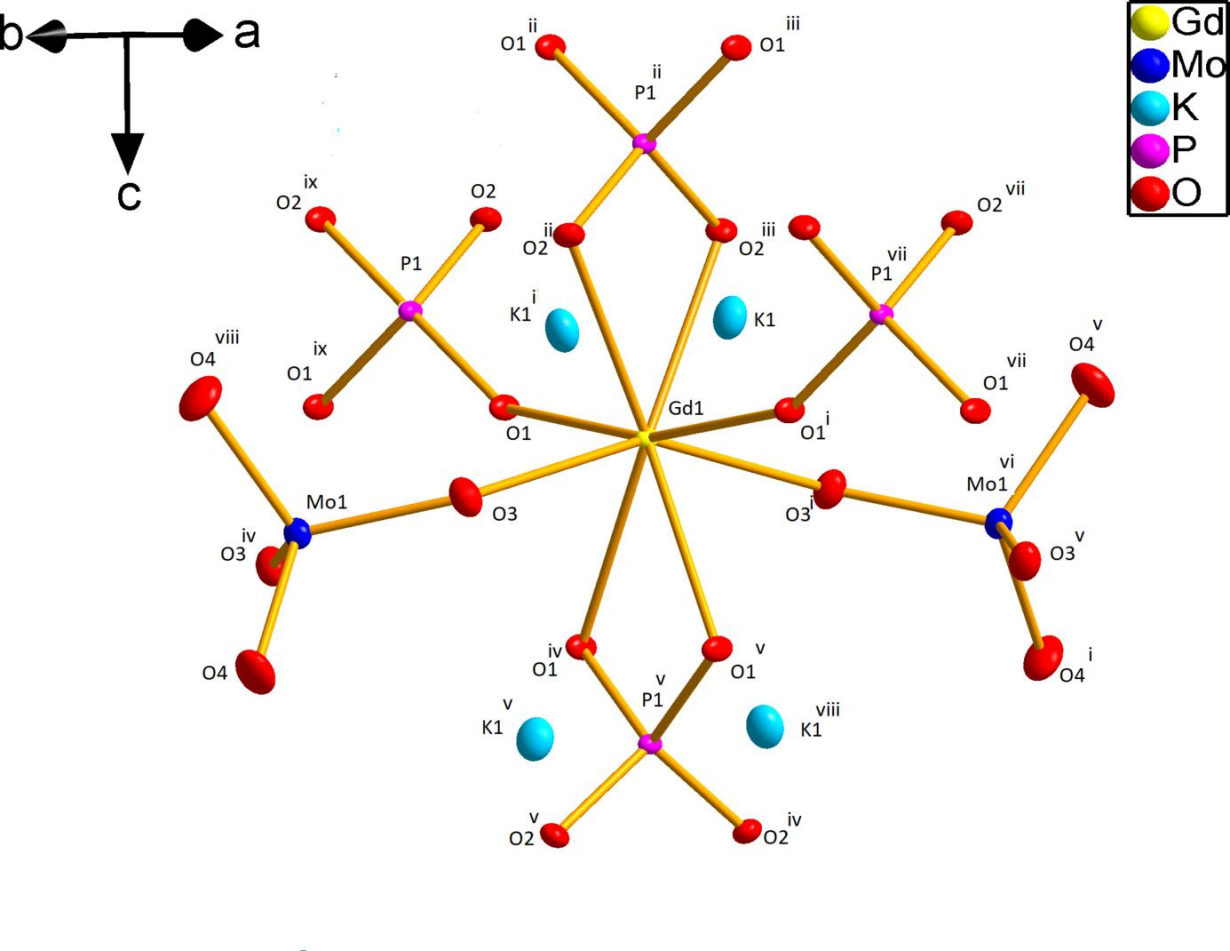
Representation of the coordination environment of gadolinium atoms in K_2_Gd(PO_4_)(MoO_4_). Displacement ellipsoids are drawn at the 50% probability level. [Symmetry codes: (i) 2 − *x*, 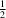 − *y*, *z*; (ii) 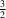 − *x*, 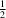 − *y*, 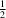 − *z*; (iii) 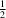 + *x*, *y*, 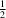 − *z*; (iv) 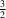 − *x*, *y*, 1 − *z*; (v) 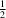 + *x*, 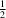 − *x*, 1 − *z*; (vii) 1 + *x*, *y*, *z*; (viii) 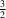 − *x*, *y*,1 − *z*; (ix) 1 − *x*, 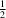 − *y*, *z*.]

**Figure 3:**
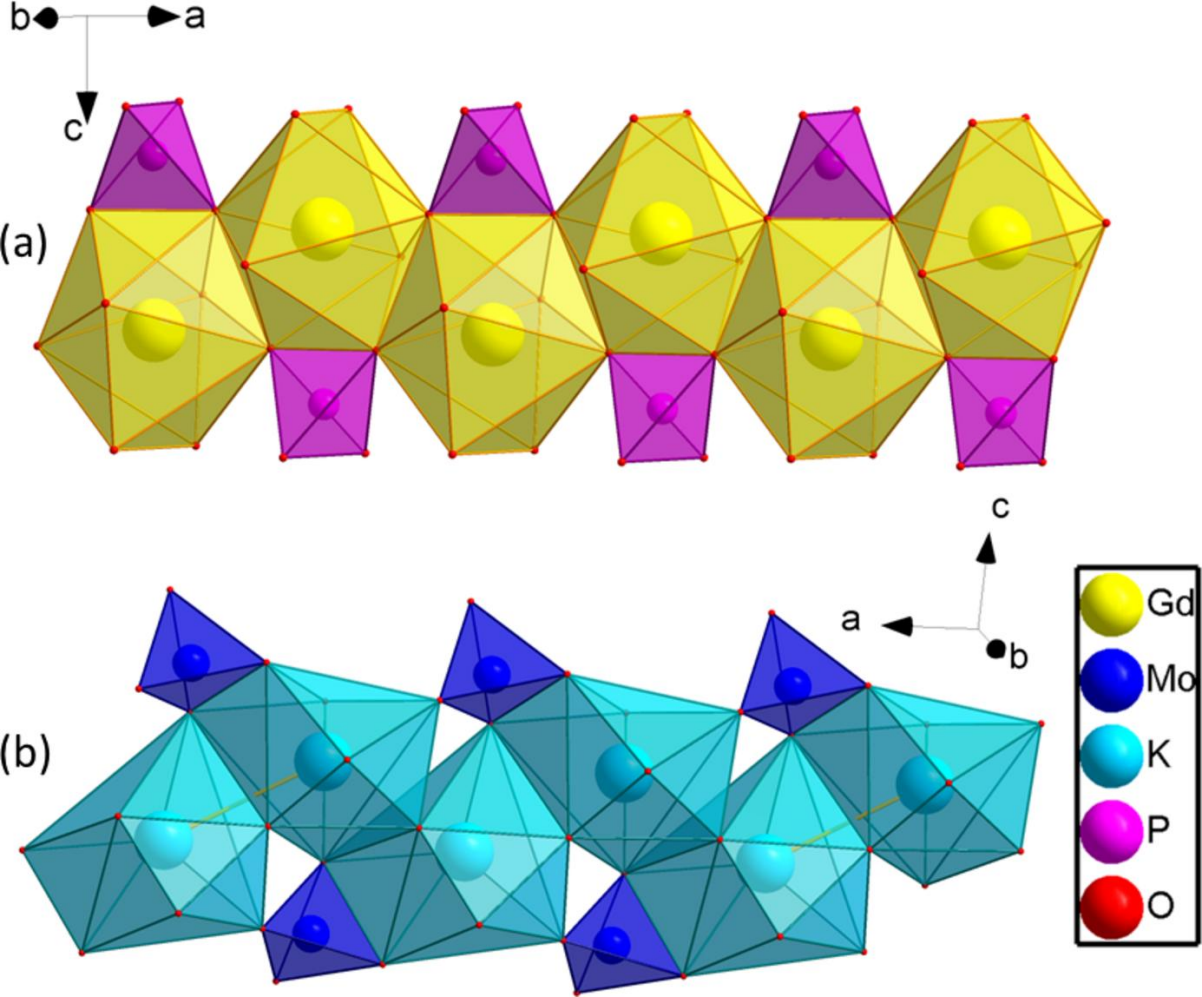
Zigzag chains build up from (*a*) GdO_8_ and (*b*) KO_8_ polyhedra

The three-dimensional framework of the title compound is organized by linking together slightly distorted GdO_8_ dodeca­hedra with non-condensed phosphate and molybdate tetra­hedra (Fig. 1 ▸). These moieties are arranged into layers perpendicular to the [010] direction with each phosphate layer being followed by two molybdate layers. In this packing, the gadolinium and potassium cations are eightfold coordinated by oxygen (Fig. 2 ▸) and ordered into zigzag chains (Fig. 3 ▸).

Each Gd cation is surrounded by two molybdate tetra­hedra and four phosphate tetra­hedra; two of the phosphate groups are coordinated in a bidentate manner (Fig. 2 ▸). The Gd—O bond lengths lie in the range 2.314 (3)–2.453 (3) Å. Among the Gd—O bond lengths, those corresponding to the bidentately coordinated phosphate groups are the longest [2.427 (2) and 2.453 (2) Å]. The chains built up from GdO_8_ polyhedra are inter­linked by phosphate moieties into [GdPO_4_] layers propagating in the *ac* plane. The nearest Gd⋯Gd distance within a zigzag chain is 3.9332 (2) Å. [Gd(PO_4_)(MoO_4_)] nets are formed by adhesion of [GdPO_4_] layers and MoO_4_ tetra­hedra above and below these layers (Fig. 1 ▸).

Both the phosphate and molybdate tetra­hedra have an almost regular geometry with typical bond lengths. The central atoms of the GdO_8_, MoO_4_ and PO_4_ polyhedra are located on a twofold axis. The potassium cation resides inside the inter­layer space having eightfold coordination, as has been found for other potassium-based representatives of this family (Zatovsky *et al.*, 2006 ▸). Importantly, there is a difference in the nearest oxygen coordination of sodium- and potassium-based frameworks. In case of Na_2_Y(PO_4_)(WO_4_), the NaO_6_ sodium environment is described as an effective 3 + 3 coordination indicating a relatively large void between two successive [Y(PO_4_)(WO_4_)] layers (Daub *et al.*, 2012 ▸).

## 3. Coordination environment calculations

The distortions of the coordination environment of gadolin­ium, potassium, phospho­rus and molybdenum have been calculated by the continuous shape measurement method with the *Shape 2.*1 program (Llunell *et al.*, 2013 ▸). The shape measurements in this work are taken from normalized coord­ination polyhedra (Alvarez, 2021 ▸). There are two types of polyhedra within the structure studied: two are tetra­hedral, namely, MoO_4_ and PO_4_ and two are eightfold coordinated, KO_8_ and GdO_8_. The shape measurements of a set of atoms with respect to a reference shape (*e.g*., the tetra­hedron, abbreviated T-4 by IUPAC) calibrates the overall distance of the atoms to the vertices of the tetra­hedral shape in the same position. Thus, a zero-shape measurement for a set of atoms indicates that the polyhedron has exactly the reference shape, expressed as *S*(T-4) = 0.00 for an ideal tetra­hedron. Increasing values of the shape measurement will be found for more distorted polyhedra, in other words, these values are essentially spatial distance minima of the central atom from a minimization polyhedral fitting procedure. For the title compound, the MoO_4_ tetra­hedron has minor distortions, as indicated by the value of *S* of 0.053. In contrast, the PO_4_ tetra­hedron reveals more severe deviations, having *C*_2_ site symmetry with a calculated value of *S* = 0.238.

In case of GdO_8_, the lowest value of *S* of 2.725 was obtained for a triangular dodeca­hedron (TDD-8) (Casanova *et al.* 2005 ▸) and KO_8_ is best described as as biaugmented trigonal prism, as indicated by the value of *S* of 3.999. Thus, the GdO_8_ polyhedron in K_2_Bi(PO_4_)(MoO_4_) is found to be a triangular dodeca­hedron (TDD-8), as has also been observed for K_2_Eu(PO_4_)(WO_4_) (Terebilenko *et al.*, 2022 ▸).

## 4. Synthesis and crystallization

Single crystals of the title compound were grown from molten salts 7K_2_Mo_2_O_7_–3K_4_P_2_O_7_ containing 5% mol of GdF_3_. A mixture of K_2_Mo_2_O_7_ and K_4_P_2_O_7_ was heated in a platinum crucible up to 1273 K. After melting, 5% mol of GdF_3_ was added to the initial molten salts under stirring. The mixture was then held at this temperature for 2 h and cooled down to room temperature at a rate of 50 K h^−1^. The solidified melt was leached out with warm water to dissolve the superfluous flux. The final product consisted of colourless plates. The yield was 64% by Gd.

## 5. Refinement

Crystal data, data collection and structure refinement details are summarized in Table 1 ▸.

## Supplementary Material

Crystal structure: contains datablock(s) I. DOI: 10.1107/S2056989023011106/oi2002sup1.cif

Structure factors: contains datablock(s) I. DOI: 10.1107/S2056989023011106/oi2002Isup3.hkl

CCDC reference: 2322198

Additional supporting information:  crystallographic information; 3D view; checkCIF report

## Figures and Tables

**Table 1:** Experimental details

Crystal data	
Chemical formula	K_2_Gd(PO_4_)(MoO_4_)
*M*_r_	490.36
Crystal system, space group	Orthorhombic, *I* *b* *c* *a*
Temperature (K)	200
*a*, *b*, *c* (Å)	6.9527 (2), 19.7112 (6), 12.2466 (3)
*V* (Å^3^)	1678.35 (8)
*Z*	8
Radiation type	Mo *K*α
μ (mm^−1^)	10.52
Crystal size (mm)	0.10 × 0.08 × 0.02

Data collection	
Diffractometer	XtaLAB Synergy, Dualflex, HyPix
Absorption correction	Gaussian (*CrysAlis PRO*; Rigaku OD, 2020 ▸)
*T*_min_, *T*_max_	0.422, 1.000
No. of measured, independent and observed [*I* > 2σ(*I*)] reflections	6547, 1079, 999
*R*_int_	0.026
(sin θ/λ)_max_ (Å^−1^)	0.707

Refinement	
*R*[*F* ^2^ > 2σ(*F* ^2^)], *wR*(*F* ^2^), *S*	0.017, 0.045, 1.13
No. of reflections	1079
No. of parameters	61
Δρ_max_, Δρ_min_ (e Å^−3^)	1.53, −0.64

Computer programs: *CrysAlis PRO* (Rigaku OD, 2020 ▸), *SHELXT2018/2* (Sheldrick, 2015*a* ▸), *SHELXL2018/3* (Sheldrick, 2015*b* ▸) and *OLEX2* (Dolomanov *et al.*, 2009 ▸).

## supplementary crystallographic information

### Crystal data

**Table:**

K_2_Gd(PO_4_)(MoO_4_)	*D*_x_ = 3.881 Mg m^−^^3^
*M**_r_* = 490.36	Mo *K*α radiation, λ = 0.71073 Å
Orthorhombic, *I**b**c**a*	Cell parameters from 4624 reflections
*a* = 6.9527 (2) Å	θ = 3.3–30.0°
*b* = 19.7112 (6) Å	µ = 10.52 mm^−^^1^
*c* = 12.2466 (3) Å	*T* = 200 K
*V* = 1678.35 (8) Å^3^	Plate, clear light colourless
*Z* = 8	0.10 × 0.08 × 0.02 mm
*F*(000) = 1784	

### Data collection

**Table:**

XtaLAB Synergy, Dualflex, HyPix diffractometer	1079 independent reflections
Radiation source: micro-focus sealed X-ray tube, PhotonJet (Mo) X-ray Source	999 reflections with *I* > 2σ(*I*)
Mirror monochromator	*R*_int_ = 0.026
Detector resolution: 10 pixels mm^-1^	θ_max_ = 30.2°, θ_min_ = 3.3°
ω scans	*h* = −8→8
Absorption correction: gaussian (CrysAlisPro; Rigaku OD, 2020)	*k* = −26→26
*T*_min_ = 0.422, *T*_max_ = 1.000	*l* = −16→16
6547 measured reflections	

### Refinement

**Table:**

Refinement on *F*^2^	0 restraints
Least-squares matrix: full	Primary atom site location: dual
*R*[*F*^2^ > 2σ(*F*^2^)] = 0.017	Secondary atom site location: difference Fourier map
*wR*(*F*^2^) = 0.045	*w* = 1/[σ^2^(*F*_o_^2^) + (0.0204*P*)^2^ + 6.0211*P*] where *P* = (*F*_o_^2^ + 2*F*_c_^2^)/3
*S* = 1.13	(Δ/σ)_max_ < 0.001
1079 reflections	Δρ_max_ = 1.53 e Å^−^^3^
61 parameters	Δρ_min_ = −0.64 e Å^−^^3^

### Special details

**Table:**

Geometry. All esds (except the esd in the dihedral angle between two l.s. planes) are estimated using the full covariance matrix. The cell esds are taken into account individually in the estimation of esds in distances, angles and torsion angles; correlations between esds in cell parameters are only used when they are defined by crystal symmetry. An approximate (isotropic) treatment of cell esds is used for estimating esds involving l.s. planes.

### Fractional atomic coordinates and isotropic or equivalent isotropic displacement parameters (Å^2^)

**Table:**

	*x*	*y*	*z*	*U*_iso_*/*U*_eq_	
Gd1	1.000000	0.250000	0.42488 (2)	0.00554 (8)	
Mo1	0.750000	0.41682 (2)	0.500000	0.00954 (10)	
K1	0.71711 (11)	0.09429 (4)	0.32974 (5)	0.01672 (16)	
P1	0.500000	0.250000	0.32047 (8)	0.0060 (2)	
O1	0.6709 (3)	0.24105 (10)	0.40045 (17)	0.0095 (4)	
O2	0.4787 (3)	0.18814 (11)	0.24608 (17)	0.0094 (4)	
O3	0.9564 (3)	0.36581 (11)	0.47067 (18)	0.0139 (4)	
O4	0.8056 (4)	0.46677 (12)	0.61376 (19)	0.0204 (5)	

### Atomic displacement parameters (Å^2^)

**Table:**

	*U*^11^	*U*^22^	*U*^33^	*U*^12^	*U*^13^	*U*^23^
Gd1	0.00341 (13)	0.00828 (12)	0.00493 (11)	−0.00007 (6)	0.000	0.000
Mo1	0.0102 (2)	0.00749 (16)	0.01089 (17)	0.000	0.00090 (13)	0.000
K1	0.0158 (4)	0.0130 (3)	0.0213 (3)	0.0014 (3)	−0.0007 (3)	0.0027 (2)
P1	0.0038 (6)	0.0093 (5)	0.0047 (5)	0.0000 (3)	0.000	0.000
O1	0.0037 (11)	0.0172 (10)	0.0076 (9)	0.0001 (8)	0.0006 (8)	0.0003 (8)
O2	0.0101 (11)	0.0110 (10)	0.0070 (9)	−0.0009 (8)	−0.0016 (7)	−0.0012 (8)
O3	0.0129 (11)	0.0111 (10)	0.0178 (11)	−0.0004 (9)	0.0034 (9)	−0.0019 (9)
O4	0.0209 (13)	0.0177 (11)	0.0226 (12)	0.0014 (10)	−0.0022 (10)	−0.0108 (10)

### Geometric parameters (Å, º)

**Table:**

Gd1—O1	2.314 (2)	K1—O1	3.037 (2)
Gd1—O1^i^	2.314 (2)	K1—O2	2.687 (2)
Gd1—O1^ii^	2.453 (2)	K1—O2^iv^	2.755 (2)
Gd1—O1^iii^	2.453 (2)	K1—O3^i^	2.958 (2)
Gd1—O2^iv^	2.427 (2)	K1—O3^vi^	3.143 (2)
Gd1—O2^v^	2.427 (2)	K1—O4^vii^	2.970 (3)
Gd1—O3	2.370 (2)	K1—O4^viii^	2.679 (2)
Gd1—O3^i^	2.370 (2)	K1—O4^vi^	3.180 (3)
Mo1—O3^ii^	1.788 (2)	P1—O1	1.550 (2)
Mo1—O3	1.788 (2)	P1—O1^ix^	1.550 (2)
Mo1—O4	1.749 (2)	P1—O2^ix^	1.529 (2)
Mo1—O4^ii^	1.749 (2)	P1—O2	1.529 (2)
			
O1—Gd1—O1^iii^	126.66 (6)	O4^ii^—Mo1—O4	111.50 (16)
O1^i^—Gd1—O1^iii^	68.18 (8)	O1—K1—O3^vi^	58.59 (6)
O1—Gd1—O1^i^	165.14 (10)	O1—K1—O4^vi^	101.73 (6)
O1^iii^—Gd1—O1^ii^	58.64 (10)	O2—K1—O2^iv^	79.43 (6)
O1^i^—Gd1—O1^ii^	126.66 (6)	O2^iv^—K1—O3^i^	60.76 (6)
O1—Gd1—O1^ii^	68.18 (8)	O2^iv^—K1—O3^vi^	118.34 (6)
O1^i^—Gd1—O2^v^	77.86 (7)	O2—K1—O3^vi^	76.61 (6)
O1—Gd1—O2^iv^	77.86 (7)	O2—K1—O3^i^	120.86 (7)
O1^i^—Gd1—O2^iv^	89.27 (7)	O2—K1—O4^vi^	77.78 (6)
O1—Gd1—O2^v^	89.27 (7)	O2^iv^—K1—O4^vi^	157.11 (7)
O1—Gd1—O3	88.71 (8)	O2^iv^—K1—O4^vii^	80.51 (7)
O1^i^—Gd1—O3	94.81 (8)	O2—K1—O4^vii^	93.86 (7)
O1^i^—Gd1—O3^i^	88.71 (8)	O3^i^—K1—O1	70.21 (6)
O1—Gd1—O3^i^	94.81 (8)	O3^i^—K1—O3^vi^	85.55 (7)
O2^v^—Gd1—O1^ii^	144.86 (7)	O3^vi^—K1—O4^vi^	53.60 (6)
O2^v^—Gd1—O1^iii^	133.34 (7)	O3^i^—K1—O4^vii^	117.92 (7)
O2^iv^—Gd1—O1^iii^	144.86 (7)	O3^i^—K1—O4^vi^	131.61 (7)
O2^iv^—Gd1—O1^ii^	133.34 (7)	O4^vii^—K1—O1	131.43 (6)
O2^iv^—Gd1—O2^v^	60.80 (10)	O4^viii^—K1—O1	147.62 (7)
O3^i^—Gd1—O1^ii^	77.67 (7)	O4^viii^—K1—O2^iv^	124.43 (7)
O3—Gd1—O1^iii^	77.67 (7)	O4^viii^—K1—O2	152.41 (7)
O3^i^—Gd1—O1^iii^	78.52 (7)	O4^viii^—K1—O3^vi^	99.58 (7)
O3—Gd1—O1^ii^	78.52 (7)	O4^viii^—K1—O3^i^	85.55 (7)
O3—Gd1—O2^v^	74.22 (7)	O4^vii^—K1—O3^vi^	155.97 (7)
O3^i^—Gd1—O2^iv^	74.22 (7)	O4^vii^—K1—O4^vi^	103.12 (7)
O3^i^—Gd1—O2^v^	132.85 (7)	O4^viii^—K1—O4^vii^	78.60 (7)
O3—Gd1—O2^iv^	132.85 (7)	O4^viii^—K1—O4^vi^	78.20 (5)
O3^i^—Gd1—O3	152.63 (11)	O1^ix^—P1—O1	101.63 (17)
O3—Mo1—O3^ii^	111.59 (14)	O2—P1—O1	111.11 (11)
O4—Mo1—O3	107.39 (11)	O2^ix^—P1—O1^ix^	111.11 (11)
O4—Mo1—O3^ii^	109.51 (11)	O2—P1—O1^ix^	113.12 (11)
O4^ii^—Mo1—O3	109.50 (11)	O2^ix^—P1—O1	113.12 (11)
O4^ii^—Mo1—O3^ii^	107.39 (11)	O2^ix^—P1—O2	106.87 (17)
			
O1^ix^—P1—O1—Gd1	−156.6 (3)	O2^ix^—P1—O2—K1	133.88 (11)
O1^ix^—P1—O1—Gd1^ii^	−0.001 (1)	O2^ix^—P1—O2—K1^x^	−106.9 (2)
O1^ix^—P1—O1—K1	112.05 (8)	O2^ix^—P1—O2—P1^ix^	0 (100)
O1^ix^—P1—O1—P1^ix^	0 (100)	O3^ii^—Mo1—O3—Gd1	15.95 (10)
O1—P1—O2—Gd1^v^	−123.85 (10)	O3^ii^—Mo1—O3—K1^iii^	−114.91 (9)
O1^ix^—P1—O2—Gd1^v^	122.59 (11)	O3^ii^—Mo1—O3—K1^i^	152.13 (15)
O1^ix^—P1—O2—K1^x^	15.7 (2)	O3^ii^—Mo1—O4—K1^xi^	32.18 (14)
O1—P1—O2—K1^x^	129.30 (18)	O3—Mo1—O4—K1^xii^	143.40 (15)
O1^ix^—P1—O2—K1	−103.53 (11)	O3^ii^—Mo1—O4—K1^xii^	−95.27 (17)
O1—P1—O2—K1	10.03 (14)	O3—Mo1—O4—K1^xi^	−89.15 (12)
O1—P1—O2—P1^ix^	0 (78)	O3^ii^—Mo1—O4—K1^iii^	116.29 (10)
O1^ix^—P1—O2—P1^ix^	0 (100)	O3—Mo1—O4—K1^iii^	−5.05 (11)
O2—P1—O1—Gd1^ii^	−120.61 (11)	O4^ii^—Mo1—O3—Gd1	−102.81 (16)
O2^ix^—P1—O1—Gd1	−37.5 (3)	O4—Mo1—O3—Gd1	135.96 (16)
O2^ix^—P1—O1—Gd1^ii^	119.18 (11)	O4—Mo1—O3—K1^i^	−87.85 (14)
O2—P1—O1—Gd1	82.7 (2)	O4^ii^—Mo1—O3—K1^iii^	126.33 (10)
O2^ix^—P1—O1—K1	−128.76 (10)	O4^ii^—Mo1—O3—K1^i^	33.37 (16)
O2—P1—O1—K1	−8.56 (12)	O4—Mo1—O3—K1^iii^	5.11 (11)
O2—P1—O1—P1^ix^	0 (100)	O4^ii^—Mo1—O4—K1^xi^	150.89 (14)
O2^ix^—P1—O1—P1^ix^	0 (23)	O4^ii^—Mo1—O4—K1^iii^	−125.01 (10)
O2^ix^—P1—O2—Gd1^v^	0.000 (1)	O4^ii^—Mo1—O4—K1^xii^	23.44 (10)

Symmetry codes: (i) −*x*+2, −*y*+1/2, *z*; (ii) −*x*+3/2, *y*, −*z*+1; (iii) *x*+1/2, −*y*+1/2, −*z*+1; (iv) *x*+1/2, *y*, −*z*+1/2; (v) −*x*+3/2, −*y*+1/2, −*z*+1/2; (vi) *x*−1/2, −*y*+1/2, −*z*+1; (vii) *x*, −*y*+1/2, *z*−1/2; (viii) *x*, *y*−1/2, −*z*+1; (ix) −*x*+1, −*y*+1/2, *z*; (x) *x*−1/2, *y*, −*z*+1/2; (xi) *x*, −*y*+1/2, *z*+1/2; (xii) *x*, *y*+1/2, −*z*+1.
